# A standardized approach to empirically define reliable assignment thresholds and appropriate management categories in deeply introgressed populations

**DOI:** 10.1038/s41598-020-59521-2

**Published:** 2020-02-18

**Authors:** Romolo Caniglia, Marco Galaverni, Edoardo Velli, Federica Mattucci, Antonio Canu, Marco Apollonio, Nadia Mucci, Massimo Scandura, Elena Fabbri

**Affiliations:** 1 Unit for Conservation Genetics (BIO-CGE), Italian Institute for Environmental Protection and Research (ISPRA), Ozzano dell’ Emilia, Bologna, Italy; 2 Conservation Unit, WWF Italia, Rome, Italy; 3 Department of Veterinary Medicine, University of Sassari, Sassari, Italy

**Keywords:** Conservation biology, Genetic hybridization

## Abstract

Anthropogenic hybridization is recognized as a major threat to the long-term survival of natural populations. While identifying F1 hybrids might be simple, the detection of older admixed individuals is far from trivial and it is still debated whether they should be targets of management. Examples of anthropogenic hybridization have been described between wolves and domestic dogs, with numerous cases detected in the Italian wolf population. After selecting appropriate wild and domestic reference populations, we used empirical and simulated 39-autosomal microsatellite genotypes, Bayesian assignment and performance analyses to develop a workflow to detect different levels of wolf *x* dog admixture. Membership proportions to the wild cluster (*q*_iw_) and performance indexes identified two *q*-thresholds which allowed to efficiently classify the analysed genotypes into three assignment classes: pure (with no or negligible domestic ancestry), older admixed (with a marginal domestic ancestry) and recent admixed (with a clearly detectable domestic ancestry) animals. Based on their potential to spread domestic variants, such classes were used to define three corresponding management categories: operational pure, introgressed and operational hybrid individuals. Our multiple-criteria approach can help wildlife managers and decision makers in more efficiently targeting the available resources for the long-term conservation of species threatened by anthropogenic hybridization.

## Introduction

Over the last decades, thanks to the growing availability of genetic and genomic data, hybridization has been increasingly studied for its evolutionary and conservational implications on the long-term survival of the involved *taxa*^1–5^. However, while natural hybridization between closely related *taxa* is frequently acknowledged as an evolutionary process providing novel adaptive gene assemblages^6,7^, anthropogenic hybridization (AH), mainly caused by intentional admixture, translocations, habitat modifications and climate changes^8–10^, is globally considered a serious conservation threat to the genetic integrity of local populations, which might be compromised by gene introgression from alien or domesticated species^11–16^. Thus, the consequences of such human-mediated process should be continuously monitored to evaluate their real effects on the viability of natural populations^5,17^.

However, to date, even in the era of genomics^4,18^, the concept of hybrid itself is rather fleeting and, consequently, legal status and management of hybrids are often poorly regulated by national and international laws, hampering the conservation of endangered species^12,14,19–22^ (for details about AH terms and definitions used through the paper see the Table 1 and the Supplementary Table S1a).

**Table 1:** Glossary of the terms and corresponding definitions used in the paper, referred to the three proposed assignment classes and management categories (with possible management priorities) in which both tested 1200 simulated and real 569 canid individual genotypes could be classified.

Assignment category	Definition	Simulated classes observed in the assignment category	Management category	Management priority
Pure individuals	Group including actually pure individuals and old admixed individuals not diagnosable or distinguishable from pure individuals [*q*_iw_ ≥ 0.995]	PW, BC8W, BC7W, BC6W, BC5W, BC4W, BC3W, BC2W	Operational pure individuals	No actions
Older admixed individuals	Group including older backcrossed individuals with marginal domestic ancestry, not diagnosable as recently admixed but with lower assignment values than wild reference individuals [0.955 ≤ *q*_iw_ < 0.995]	BC7W, BC6W, BC5W, BC4W, BC3W, BC2W	Introgressed individuals	Low priority actions
Recent admixed individuals	Group including mostly recent admixed individuals and a small fraction of older admixed individuals [*q*_iw_ < 0.955]	F2, F1, BC1W, BC2W	Operational hybrids	High priority actions

Assignment classes are based on individual 39-STR q_iw_-values and applying the two selected *q*-thresholds (0.995, representing the minimum individual *q*_iw_ assignment value of the simulated and real wild parentals (see Table 2), and 0.955, selected on the basis of the performance analysis (see Supplementary Table S2)). Bayesian assignment analyses were performed by the software Parallel Structure, assuming *K* = 2 clusters and using the “*Admixture*” and “*Independent allele frequencies*” models. Simulated classes include: wild (PW) and domestic (PD) parentals, first (F1) and second (F2) generation hybrids, and eight backcross generations (BC1W-BC8W) with wild parentals.

The management of individuals originated from AH is still debated^5,16^, especially since it is not always clear whether the removal of admixed individuals (Supplementary Table S1a) might represent an appropriate and feasible conservation strategy^3,16,21,23,24^, or which methods should be applied for their control (e.g. sterilization, captivation, lethal removal). Finally, it is still uncertain whether admixed individuals originated from hybridization events that occurred several generations in the past should be targets of management actions^10,16,24–26^, especially when they retain likely adaptive variants (e.g. the black coat in wolves^27^ and the domestic goat MHC haplotypes in the Alpine ibex^28^).

However, solving these doubts is far from trivial. In fact, while identifying first generation (F1) hybrids might be relatively simple, recognizing admixed individuals originated from recurrent or ancient hybridization events, as well as different ranges of generations of admixture, is a demanding or a virtually impossible task^5,11^. Thus, it would be highly beneficial to introduce standardized criteria to classify admixed individuals based on their actual level of risk to spread alien or domestic variants, allowing to prioritize management actions, more efficiently allocate efforts and resources, and support long-term conservation plans^3,5,14,23^.

Nevertheless, the detection of hybrids and backcrosses (Supplementary Table S1a) can be further complicated by a number of technical or statistical issues. Many hybridizing *taxa* are elusive and difficult to detect, therefore hybridization monitoring programs are usually carried out relying on opportunistically-collected and often degraded biological materials^23,29–37^. These approaches limit the use of more diagnostic large panels of markers such as Single Nucleotide Polymorphisms (SNPs) and the employment of more than a few tens of microsatellites (or STRs, Short Tandem Repeats), which generally allow the detection of hybridization events occurred only up to two or three generations in the past^5,38,39^. Another critical point is represented by the selection of appropriate reference populations to use in the assignment analyses, that should include a sufficient number of pure individuals representative of the genetic variability of the analysed *taxa*. Furthermore, particular attention should be reserved to the choice of adequate statistical computations for the assignment procedures, in order to minimise the risk that individual assignment probabilities might be affected by the allele frequencies of other samples to be assigned. In addition, the selection of statistically supported assignment *q-*thresholds based on individual assignment (*q*_i_-values) extrapolated from simulated data is necessary to ensure the realistic and reliable identification of admixed individuals^5,16,40–42^.

Therefore, using both empirical and simulated data, in this study we developed a simple but effective workflow (Supplementary Fig. S1) to overcome some of the aforementioned issues and to reliably detect different levels of introgression^43^, maximizing the identification of pure and admixed individuals, either hybrids or backcrosses (Supplementary Table S1a). We then tested this approach on an interesting example of AH^16,26^, taking place between the wolf (*Canis lupus*) and its domestic counterpart, the dog (*C. l. familiaris*). In particular, we investigated samples from the Italian wolf population (*C. l. italicus*), which experienced a demographic scenario characterized by protracted isolation south of the Alps and recurrent bottlenecks that made Italian wolves sharply genetically differentiated from any other wolf population^4,24,26,44–47^. Although the Italian wolf population is now recovering thanks to legal protection and the increased availability of suitable habitats and prey^48^, it is still threatened by accidental or illegal killings^49,50^, but also by anthropogenic hybridization, as repeatedly documented by genetic and genomic data showing gene flow from the domestic to the wild subspecies^24–26,31,33,37,51,52^.

Specifically, we used this case-study to:

1. delineate strict criteria for choosing the most appropriate reference parental populations;
2. based on simulated data, determine adequate and reliable assignment *q-*thresholds to consistently classify individuals into discrete levels of domestic ancestry;
3. apply a standardized and stable Bayesian method to probabilistically assign unknown individuals to one of the ancestry classes;
4. accordingly, define appropriate management categories to prioritize possible mitigation actions.

## Results

### Selection of the reference populations

Following strict morphological, genetic and genomic criteria for sample selection (see Materials and Methods), we retained from the ISPRA (Italian Institute for Environmental Protection and Research) canid database the genotypes of 190 wolves and 89 dogs typed at a panel of 39 STRs commonly used to reconstruct individual genotypes in some of the most recent studies on wolf *x* dog hybridization in Europe^26,33,47,53^. Selected individuals showed no missing data nor the occurrence of allelic dropout and false alleles, thus they were assumed as reference genotypes in the downstream analyses and were used in HybridLab^54^ to simulate 100 genotypes of wild (PW) and domestic (PD) parentals, first (F1) and second (F2) generation hybrids, and eight backcross generations (BC1W-BC8W) with wild parentals (Supplementary Table S1b).

### Evaluation of the relative reliability and replicability of the Bayesian approaches

Results from the four independent Bayesian clustering runs obtained analysing the 1,479 canid individuals (including both reference and simulated genotypes) at *K* = 2 with the *A* and *I* models showed that the “one-by-one” assignment method, implemented in Parallel Structure, confirmed to be highly stable^55^, with an average variation of only 0.0074 (±0.0085 SD) in individual coefficient values (*q*_i_) among runs. This low variation allowed us to present outcomes from the first run, without the need to condense the results from multiple runs as it is usually needed when dealing with larger variations.

### Membership proportions and individual coefficients from the assignment tests

**Figure 1:**
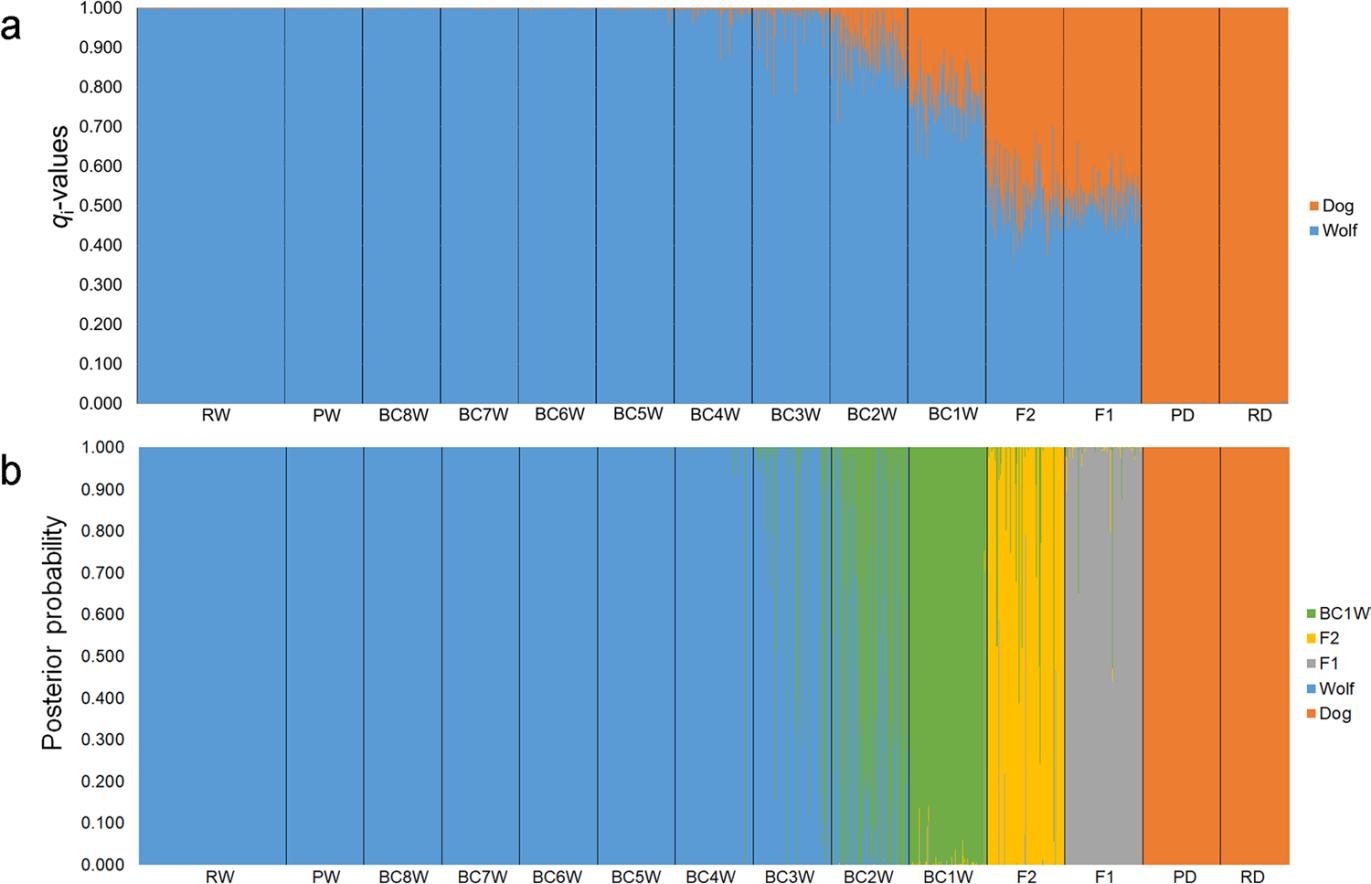
(**a**) Bar plotting of the individual *q*_i_-values obtained assigning the 39-STR genotypes of the simulated wild (PW) and domestic (PD) parentals, first (F1) and second (F2) generation hybrids, and eight backcross generations (BC1W-BC8W) with wild parentals to the wolf (RW) and dog (RD) reference populations. Each individual is represented by a vertical line partitioned into colored segments, whose length is proportional to the individual coefficients of membership (*q*_i_) to the wolf and dog clusters inferred by a Bayesian assignment analyses performed by the software Parallel Structure, assuming *K* = 2 clusters and using the “*Admixture*” and “*Independent allele frequencies*” models. (**b**) Posterior probabilities estimated, for the 39-STR genotypes of the simulated wild (PW) and domestic (PD) parentals, first (F1) and second (F2) generation hybrids, and eight backcross generations (BC1W-BC8W) with wild parentals to the wolf (RW) and dog (RD) reference populations, using the software NewHybrids with the “*Jeffreys-like*” priors for both mixing proportions and allele frequencies. Each individual is represented by a horizontal bar divided in five segments corresponding to its probability to belong to five classes: wild and domestic parentals (PW and PD), F1, F2, and first backcrosses of F1 with wolves (BC1W).

We used the assignment results produced by Parallel Structure^55^ to estimate the average membership proportions (*Q*_i_) and individual coefficients (*q*_i_) of each predefined group (Fig. 1 and Table 1). We also estimated 90% credibility intervals (CI) for both *Q*_i_ and *q*_i_.

All the wild reference individuals were probabilistically assigned to the same cluster I with *Q*_w_ = 0.999 (CI = 0.996–1.000), and with individual *q*_iw_ ranging from 0.995 (CI = 0.966–1.000) to 1.000 (CI = 0.998–1.000). All the domestic reference individuals were assigned to the same cluster II with *Q*_d_ = 0.998 (CI = 0.991–1.000), and with domestic *q*_id_ ranging between 0.993 (CI = 0.949–1.000) and 1.000 (CI = 0.998–1.000) (Fig. 1a and Table 2).

**Table 2:** Parallel Structure columns enclose average membership proportions *Q*_i_ to the wolf (*Q*_wolf_) cluster with their confidence intervals (90% CI) and ranges of the individual assignment coefficients *q*_i_ to the wolf (*q*_wolf_) with their credibility intervals (90% CI) estimated through the Bayesian assignment analyses of the 39-STR reference and simulated genotypes performed in Parallel Structure, assuming *K* = 2 clusters and using the “*Admixture*” and “*Independent allele frequencies*” models. NewHybrids columns enclose average posterior probabilities to belong to the genotype classes of domestic and wild parentals (PD and PW), first (F1) and second (F2) generation hybrids, and first backcrosses of F1 with wolves (BC1W) as inferred through the Bayesian assignment analyses of the 39-STR reference and simulated genotypes performed in NewHybrids using the “*Jeffreys-like*” priors.

Group	Parallel Structure	Parallel Structure	NewHybrids				
	Mean *Q*_wolf_ (90% CI)	Range of *q*_wolf_ (90% CI)	PD	PW	F1	F2	BC1W
RW	0.999 (0.996–1.000)	0.995 (CI = 0.966–1.000)–1.000 (CI = 0.998–1.000)	0.000	1.000	0.000	0.000	0.000
RD	0.002 (0.000–0.009)	0.007 (CI = 0.000–0.051)–0.000 (CI = 0.000–0.002)	1.000	0.000	0.000	0.000	0.000
PW	0.999 (0.997–1.000)	0.995 (CI = 0.966–1.000)–1.000 (CI = 0.998–1.000)	0.000	1.000	0.000	0.000	0.000
PD	0.002 (0.000–0.007)	0.007 (CI = 0.000–0.051)–0.000 (CI = 0.000–0.002)	1.000	0.000	0.000	0.000	0.000
F1	0.515 (0.384–0.644)	0.415 (CI = 0.282–0.551)–0.661 (CI = 0.525–0.786)	0.000	0.000	0.983	0.006	0.011
F2	0.524 (0.393–0.652)	0.357 (CI = 0.222–0.496)–0.702 (CI = 0.579–0.814)	0.000	0.000	0.022	0.922	0.056
BC1W	0.778 (0.662–0.881)	0.618 (CI = 0.491–0.737)–0.923 (CI = 0.812–1.000)	0.000	0.001	0.008	0.006	0.984
BC2W	0.907 (0.816–0.971)	0.709 (CI = 0.585–0.822)–0.999 (CI = 0.996–1.000)	0.000	0.399	0.000	0.000	0.601
BC3W	0.973 (0.921–0.997)	0.819 (CI = 0.705–0.918)–0.999 (CI = 0.998–1.000)	0.000	0.877	0.000	0.000	0.123
BC4W	0.993 (0.972–0.999)	0.815 (CI = 0.698–0.917)–1.000 (CI = 0.998–1.000)	0.000	0.988	0.000	0.000	0.012
BC5W	0.998 (0.989–1.000)	0.962 (CI = 0.849–1.000)–1.000 (CI = 0.998–1.000)	0.000	1.000	0.000	0.000	0.000
BC6W	0.999 (0.993–1.000)	0.987 (CI = 0.927–1.000)–1.000 (CI = 0.998–1.000)	0.000	1.000	0.000	0.000	0.000
BC7W	0.999 (0.996–1.000)	0.993 (CI = 0.949–1.000)–1.000 (CI = 0.998–1.000)	0.000	1.000	0.000	0.000	0.000
BC8W	0.999 (0.997–1.000)	0.997 (CI = 0.977–1.000)–1.000 (CI = 0.998–1.000)	0.000	1.000	0.000	0.000	0.000

Data comprise the 39-STR genotypes of the reference wild (RW) and reference domestic (RD) individuals, simulated wild (PW) and domestic (PD) parentals, first (F1) and second (F2) generation hybrids, and eight backcross generations (BC1W-BC8W) with wild parentals.

The wild and domestic simulated parental populations showed *Q*_i_ and *q*_i_-values almost completely overlapping with those of the wild and domestic reference populations, and were assigned to their respective clusters with *q*_iw_ ≥ 0.995 for the wild and *q*_id_ ≥ 0.989 for the domestic parentals (Fig. 1a).

Simulated F1 and F2 showed, as expected, intermediate *Q*_i_-values (F1: *Q*_w_ = 0.515, CI = 0.384–0.644; F2: *Q*_w_ = 0.524, CI = 0.393–0.652), while individual *q*_i_ were 0.415 (CI = 0.282–0.551) ≤ *q*_iw_ ≤ 0.661 (CI = 0.525–0.786) for F1 and 0.357 (CI = 0.222–0.496) ≤ *q*_iw_ ≤ 0.702 (CI = 0.579–0.814) for F2. Backcrossed genotypes showed variable *Q*_i_-values that started to completely overlap to those of the parental populations in BC6W (Fig. 1a and Table 3), though a partial overlapping (up to 7%) was observed already from BC2W (Fig. 1a).

**Table 3:** Proportions of real and simulated 39-STR genotypes correctly identified as assignment-pure, older admixed and recent admixed individuals and, consequently, classifiable as operational pure, introgressed and operational hybrid individuals applying the two selected *q*-thresholds (0.995, representing the minimum individual *q*_iw_ assignment value of the simulated and real wild parentals, (see Table 2), and 0.955, selected on the basis of the performance analysis (see Supplementary Table S2)) which, minimizing the risk of both type I and type II errors, are able to efficiently discriminate between the three proposed assignment classes and corresponding management categories.

Group	Management categories				
	Operational pure individuals		Introgressed individuals	Operational hybrids	
	Parallel Structure	NewHybrids	Parallel Structure	Parallel Structure	NewHybrids
	*q*_iw_ ≥ 0.995	PW	0.955 ≤ *q*_iw_ < 0.995	*q*_iw_ < 0.955	F1, F2, BC1W
RW	100	100	0	0	0
PW	100	100	0	0	0
F1	0	0	0	100	100
F2	0	0	0	100	100
BC1W	0	0	0	100	100
BC2W	7	40	22	71	60
BC3W	40	87	40	20	13
BC4W	76	99	21	3	1
BC5W	93	100	7	0	0
BC6W	99	100	1	0	0
BC7W	99	100	1	0	0
BC8W	100	100	0	0	0

Bayesian assignment analyses were performed by the software Parallel Structure, assuming *K* = 2 clusters and using the “*Admixture*” and “*Independent allele frequencies*” models, and by the software NewHybrids assuming five genotype classes (domestic and wild parentals (PD and PW), first (F1) and second (F2) generation hybrids, and first backcrosses of F1 with wolves (BC1W)), using the “*Jeffreys-like*” priors. Data comprise the 39-STR genotypes of the reference wild individuals (RW), simulated wild parentals (PW), first (F1) and second (F2) generation hybrids, and eight backcross generations (BC1W-BC8W) with wild parentals.

Interestingly, as expected, CI values were overall significantly negatively correlated (*r* = −0.91; *P* < 0.0001) with *q*_iw_-values (Supplementary Fig. S2a), and their mean widths in the simulated admixed individuals were on average significantly larger (*t* = 82.4, *P* < 0.0001; *t*-test) than in parental wolves (Supplementary Fig. S2a).

Bayesian clustering results obtained from Parallel Structure^55^ analysing a reduced set of 12 STRs, commonly utilized for genotyping low-content DNA samples in non-invasive genetic monitoring projects^52,56^, showed that, even though all reference parental genotypes were fully assigned to their clusters, the 12 STRs provided a lower resolution in detecting backcrosses. Indeed, 4% of BC1W showed a partial *q*_iw_ overlap to those of the wild parental population (details are described in the Supplementary Text S1).

The assignment results were robust even when simulating increasingly high levels of allelic dropout (ADO) and missing data, showing less than 2% discrepancy of the individual *q*_i_-values even with 30% ADO and missing loci at 39 STRs, and less than 4% at 12 STRs. When we considered 10% ADO and missing data, discrepancies were less than 1% at 39 STRs and less than 3% at 12 STRs.

### Selection and performance of the appropriate thresholds

**Figure 2:**
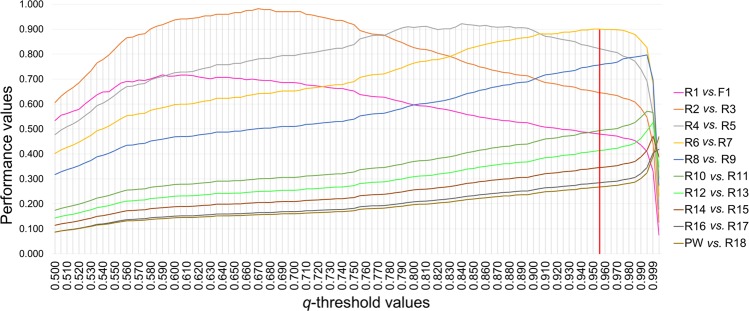
Graphical trends of the average performances (on the y-axis) estimated for increasing values of *q*-thresholds (on the x-axis). Each performance was computed as the product between the mean efficiency (the ratio of the number of admixed individuals correctly identified on the total number of admixed individuals actually included in the sample) and the accuracy (the number of admixed individuals correctly assigned to a certain admixture class on the total number of individuals actually belonging to that class) obtained considering individual *q*_i_-values of the simulated 39-STR genotypes estimated from the Bayesian assignment analyses performed in Parallel Structure, assuming *K* = 2 clusters and using the “*Admixture*” and “*Independent allele frequencies*” models. Each *q*-threshold was tested considering comparisons between groups (**Rn**) including simulated individuals for increasing levels of admixture. **R1**: PW, BC8W to BC1W & F2; **R2**: PW, BC8W to BC1W; **R3**: F1 & F2; **R4**: PW, BC8W to BC2W; **R5**: BC1W, F2 & F1; **R6**: PW, BC8W to BC3W; **R7**: BC2W to BC1W, F2 & F1; **R8**: PW, BC8W to BC4W; **R9**: BC3W to BC1W, F2 & F1; **R10**: PW, BC8W to BC5W; **R11**: BC4W to BC1W, F2 & F1; **R12**: PW, BC8W to BC6W; **R13**: BC5W to BC1W, F2 & F1; **R14**: PW, BC8W to BC7W; **R15**: BC6W to BC1W, F2 & F1; **R16**: PW & BC8W; **R17**: BC7W to BC1W, F2 & F1; **R18**: BC8W to BC1W, F2 & F1. The vertical red line identifies the *q*-threshold (*q*_i_ = 0.955) selected on the basis of the performance analysis carried out comparing **R6** *vs*. **R7** (yellow line).

**Figure 3:**
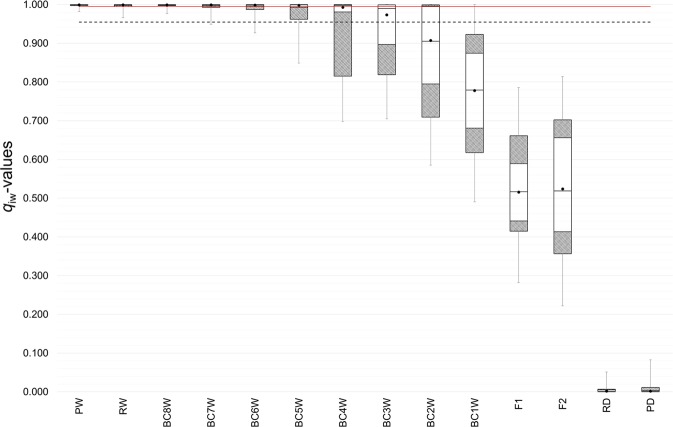
Box plots of individual *q*_iw_-values (on the y-axis) observed in parental and simulated 39-STR genotypes (on the x-axis) estimated from the Bayesian assignment analyses performed in Parallel Structure, assuming *K* = 2 clusters and using the “*Admixture*” and “*Independent allele frequencies*” models. White solid boxes include 90% of the observed data values. Dashed gray boxes contain the 5^th^ percentile and the 95^th^ percentile of the observed data values. Black dots indicate mean data values. Middle transversal lines inside boxes show median data values (the 50^th^ percentile). Box plot wiskers include the ranges of the confidence intervals. The dashed line represents the *q*-threshold of 0.955, selected on the basis of the performance analysis (see Supplementary Table S2), which, minimizing the risk of both type I and type II errors, is able to efficiently discriminate between recent admixed (F1-BC2W) and older admixed (BC3W-BC8W) individuals. The red line indicates the *q*-threshold identified at 0.995, value corresponding to the minimum individual *q*_iw_ of both simulated and real wolf parentals (see Table 2), which, minimizing the risk of both type I and type II errors, is able to efficiently discriminate between older admixed (BC3W-BC8W) and pure individuals.

Accuracy, efficiency and performance^38^ were calculated for different candidate *q*-thresholds ranging from 0.500 to 0.999 (Fig. 2 and Supplementary Table S2) and each *q*-threshold was tested between groups of simulated individuals at increasing levels of admixture (e.g. PW *vs*. BC8W to BCW1, F2 & F1; PW & BC8W *vs*. BC7W to BCW1, F2 & F1; PW, BC8W & BC7W *vs*. BC6W to BCW1, F2 & F1; PW, BC8W, BC7W & BC6W *vs*. BC5W to BCW1, F2 & F1; PW, BC8W, BC7W, BC6W & BC5W *vs*. BC4W to BCW1, F2 & F1, etc.). A performance higher than 0.90 was obtained only for three category combinations: PW & BCW8 to BCW1 *vs*. F1 & F2 (best performance = 0.982 at *q*-threshold = 0.670), PW & BCW8 to BCW2 *vs*. BC1W, F2 & F1 (best performance = 0.922 at *q*-threshold = 0.840) and PW & BCW8 to BCW3 *vs*. BC2W, BC1W, F2 & F1 (best performance = 0.900 at *q*-thresholds = 0.950–0.955). Therefore, to be as conservative as possible, we decided to retain the highest *q*-threshold value (0.955) of the latter category combination to efficiently discriminate between recent (F1-BC2W) and older admixed individuals (from BC3W onwards). This *q*-threshold was able to correctly identify a larger portion (40%) than the other highly performing *q*-thresholds (39% for a *q*-threshold = 0.950 for the same categories, 31% for a *q*-threshold = 0.840 and 20% for *q*-threshold = 0.670) of the 1000 simulated admixed genotypes, maximizing the recognition of recent admixed individuals (Fig. 3 and Table 3). At this *q*-threshold, indeed, 100% of the wolf *x* dog F1, F2, BC1W and 71% of BC2W were coherently classified as recent admixed individuals.

However, since none of the highly-performing *q*-thresholds was able to reliably discriminate between older admixed and pure individuals, we introduced a second *q*-threshold at 0.995, representing the minimum individual *q*_iw_ assignment value of the simulated and real wild parentals. Therefore, we assumed that the assignment interval in the range 0.955-0.995 could include older admixed individuals, showing only a marginal dog ancestry (<5%). In this way, 22% of BC2W were classified as older admixed individuals, together with 40% of BC3W, 21% of BC4W, 7% of BC5W, 1% of BC6W and 1% of BC7W (Table 3). Above this second *q*-threshold type I errors confirmed to be absent but type II errors were further minimized since we found 7% of BC2W, 40% BC3W, 76% BC4W, and more than 90% of BC5W-BC8W clustering together with reference and simulated wolf parentals (Table 3).

When we considered their 90% confidence intervals, their mean widths in the older admixed individuals were significantly larger than in pure wolves (*t* = 61.1, *P* < 0.0001; *t*-test) as well as they were significantly larger in recent admixed than in older admixed (*t* = 38.5, *P* < 0.0001; *t*-test) individuals (Supplementary Fig. S2b). Additionally, all individuals from BC3W to BC8W showed CI values higher than 0.955, thus representing an additional criterion to identify recent admixed individuals^5,57^.

Also when we considered the reduced 12-marker panel, we correspondingly retained two *q*-thresholds: the first value of 0.975 was chosen since it efficiently discriminated between recently admixed individuals (F1-BC2W) and all the other simulated classes, including older admixed (from BC3W onwards) plus pure (PW) individuals. A second *q*-threshold of 0.990, representing the minimum individual *q*_iw_ assignment value of the simulated and real wild parentals, was selected to reliably discriminate between older admixed and pure individuals (for details see the Supplementary Text S1).

The four replicated runs in NewHybrids^58^ showed almost identical outcomes using both “*Jeffreys-like*” (*t* ≥ 0.006, *P* ≥ 0.91; *t*-tests for all pairwise combinations) and “*Uniform*” (*t* ≥ 0.032, *P* ≥ 0.97; *t*-tests) priors, with no significant differences even between the two models (*t* = 0.45, *P* = 0.65; *t*-test between average values of the four runs), therefore we decided to present only the results from the first NewHybrids run obtained with the “*Jeffreys-like*” priors (Table 2). Overall, the Bayesian assignment performed in NewHybrids^58^ also proved to be efficient (Fig. 1b), showing proportions of real and simulated samples correctly assigned to their own categories up to BC1W, not significantly (${\chi }_{6}^{2}=1.74$, *P* = 0.94; χ^2^-test) different from the proportions achieved using Parallel Structure (Table 3), despite the very different assumptions they rely on^55,58^. All wild and domestic references and all wild and domestic simulated parentals had the best posterior probabilities (*P* ≥ 0.999) to be purebred animals (Table 2). Most F1 (98.3%), F2 (92.2%) and BC1W (98.4%) were clearly assigned to their own categories (*P* ≥ 0.900) showing posterior probabilities to belong to pure wolves or dogs always <0.001 (Fig. 1b). Interestingly and coherently with the detection power of the software^58^, looking at BC2W 60% of them were misclassified as BC1W and 40% as pure wild parentals (Table 2 and Table 3). Additionally, 88% BC3W, 99% BC4W and all the other BCW showed significant posterior probabilities (*P* ≥ 0.900) to be pure wolves (Table 2 and Table 3).

However, when we tried to extend NewHybrids assignment to classes older than BC1W (from BC2W to BC8W), results were highly different from expected since both real and simulated genotypes were never clearly attributed to their own genotypic categories, with the only exceptions of F1 and F2 individuals (Supplementary Table S3).

### Application to real data

The application of the selected *q*-thresholds and Bayesian methodology to the 39-STR real genotypes (in which neither missing data nor allelic dropout were ever detected) belonging to 569 putative wolves collected from 1987 to 2019 throughout the whole wolf distribution range in Italy^26,33,47,52^ highlighted that 12.7% of them were diagnosable as recent admixed individuals (*q*_iw_ < 0.955), that we thus assigned to the management class of operational hybrids. Another 13.5% were diagnosable as older admixed individuals (0.955 ≤ *q*_iw_ < 0.995), thus operationally classified as introgressed individuals (Table 1 and Table 4). Conversely, the remaining 73.8% of the analysed real genotypes were identified as assignment-pure wolves (*q*_iw_ ≥ 0.995), thus falling in the management class of the operational pure individuals (Table 1 and Table 4).

**Table 4:** Numbers and percentages of the 39-STR real genotypes belonging to 569 putative wolves (236 females and 333 males) correctly identified as assignment-pure, older admixed and recent admixed individuals, and, consequently classifiable as operational pure, introgressed and operational hybrid individuals.

Marker type	Management categories		
	Operational pure individuals	Introgressed individuals	Operational hybrids
	*q*_iw_ ≥ 0.995	0.955 ≤ *q*_iw_ < 0.995	*q*_iw_ < 0.955
39-STRs (N = 569)	420 (73.8%) [247 males]	77 (13.5%) [45 males]	72 (12.7%) [41 males]
Dog mtDNA CR haplotypes (N = 0/569, 0%)	0 (0%)	0 (0%)	0 (0%)
Dog Y-linked STR haplotypes (N = 61/333 males, 18.3%)	25 (10%)	15 (33.3%)	21 (51.2%)
*K*-locus 3-bp melanistic deletion (N = 32/569, 5.6%)	13 (3%)	5 (6.5%)	14 (19.7%)

Management categories were obtained applying the two selected *q*-thresholds of 0.995 and 0.955 to the individual *q*_i_-values estimated from the Bayesian assignment analyses performed in Parallel Structure, assuming *K* = 2 clusters and using the “*Admixture*” and “*Independent allele frequencies*” models. Assignments to the three proposed management categories were completed adding further percentages of dog ancestry derived from the uniparental (mtDNA control region and four Y-linked STRs) markers and from the functional melanistic deletion at the β-defensin CBD103 gene (corresponding to the *K-*locus). The percentages of individuals carrying dog-derived Y haplotypes in each management category always refer to the number of males. The possible reasons for the lower observed frequency of the *K*-locus deletion compared to dog Y haplotypes are twofold: on one side, although we do not have phenotypic information, we expect that only a portion of the dogs responsible for hybridization carried the deletion and were black coated, while all of them are expected to carry a dog-specific Y haplotype. Moreover, while in some specific environments (e.g. in Yellowstone National Park) the *K*-locus deletion could provide fitness advantages, there is no such evidence in the Italian wolf population, where traces of “resistance to introgression” for the chromosomal region hosting the *K*-locus have been actually showed^26^.

When real data assignments were completed with uniparental and coding markers (mtDNA, Y-STRs and *K-*locus), 68.0% of the analysed real genotypes did not show any traces of dog ancestry (Table 4). In particular, none of the individuals identified as operational pure animals showed dog mtDNA haplotypes, whereas 6% of them (corresponding to 10% of the pure males) showed dog Y-STR haplotypes and 3% had the melanistic 3-bp deletion (Table 4). These animals could represent additional older admixed individuals retaining domestic alleles in other genetic markers not included in the nuclear STR-based workflow. Interestingly, another 19 operational pure individuals (4.5%) showed dog-like phenotypic traits (white claws and/or spur on the hind legs^26^), which were not genetically detected.

When we applied the selected *q*-thresholds of 0.975 and 0.990 to the 12-STR genotypes of the real 569 putative wolves, the percentages of operational pure animals and of operational hybrids respectively increased to 77.% and 15.5%, whereas the percentage of introgressed individuals decreased to 7.1% since a part of them was misclassified as pure and another part was misclassified as recent admixed individuals (for details see the Supplementary Text S1).

## Discussion

While natural hybridization has been widely acknowledged as a powerful evolutionary force^6,7^, during last decades anthropogenic hybridization considerably contributed to threat the genomic integrity and survival of a number of *taxa* through the introgression of alien or domestic alleles in the gene pool of natural populations^3,11,12,15,41,42,59^. In particular, though some studies documented cases of beneficial introgression of domestic mutations in wild populations of North American wolves^27^ and Alpine ibexes^28^, introgressive hybridization with domestic forms is globally recognized as a significant risk factor for the conservation of several wild *taxa*^14,24,28,60–62^. However, though being essential to understand the real impact of the phenomenon and to design sound conservation strategies^16,23^, the identification of hybrids and their backcrosses remains far from trivial even in the genomic era^3–5,10,13^. In the common practice, the domestic ancestry of biological samples is usually assessed typing their DNA at presumably neutral molecular markers and probabilistically assigning the obtained genotypes to reference parental populations by Bayesian statistics^57,63^. Consequently, Bayesian assignment values (*q*_i_-values) are considered key parameters for management initiatives^5,16^ and well relate to genomic proportions of parental ancestry estimated by genomic approaches such as the PCA-based admixture deconvolution methods^26,64,65^.

Nonetheless, detecting admixture signals between subspecies sharing a very recent common ancestry is often hampered by the difficulty to *a priori* identify pure individuals^24,26^ and a number of pitfalls may sway the analyses, thus strict criteria should be applied for a reliable identification of admixed individuals: (1) reference parental populations should be composed by the genetic profiles of a sufficient number of individuals (e.g. at least 40 for each reference population^5^), obtained through the genotyping of high-quality samples at a large number of markers, and lacking any genetic - and possibly morphological - signature of hybrid ancestry; (2) *q*_i_-values of unclassified individuals should be estimated by assigning them to parental populations through a repeatable and standardized Bayesian statistical approach; (3) the *a posteriori* classification of individuals should be based on *q*-thresholds previously established from the distribution of *q*_i_-values observed in simulated genotypes^5,33,38^.

In this study, we implemented a rapid and efficient standardized workflow (Supplementary Fig. S1) to molecularly detect and classify different levels of admixture in individuals belonging to the Italian wolf population (*C. l. italicus*), a *taxon* in which wild *x* domestic hybridization has been repeatedly documented^24–26,31,33,37,51,66,67^.

The selection of a sufficient number of non-admixed parental individuals to use as reference populations in the assignment analyses was made possible by testing a large national database that includes hundreds of individuals sampled from the entire subspecies distribution range, which had been all formerly morphologically described and molecularly characterized at different sets of genome-wide (STRs and SNPs) markers^26,33,52^. Therefore, initiatives aiming at systematically collecting population-wide samples of target species should be strongly sustained by national or local authorities, possibly including also samples from nearby populations in order to take into account possible gene flows^22,68^ and, whenever achievable, detailed information on possible phenotypical anomalies^5,24,26^.

The simulation of hybrid and backcrossed genotypes, as well as a sufficient number of ancestry-informative markers able to discriminate even closely-related species or subspecies, is then required in order to establish reliable *q*-thresholds discriminating between different levels of admixture classes^5,18,38^.

In addition, stable statistical Bayesian approaches, such as that implemented in Parallel Structure^55^, are strongly recommended to minimize the risk of biased assignment probabilities to an *a priori* assumed number of populations^40^, which might occur when sample sizes vary among analyses or when unknown samples with variable levels of admixture (namely including both pure and admixed individuals) are analysed simultaneously instead of one by one^40,69,70^, conversely to other fully (Structure, NewHybrids, Baps) or partially (GeneClass) Bayesian assignment methods commonly applied for admixture identifications^33,41,42,71–76^. As expected, the “one-by-one” approach with Parallel Structure^55^ performed reliably, with very limited fluctuations of both *Q*_i_ and *q*_i_ among different replicates of the same runs. Up to BC1W, results were also highly concordant with the results obtained from the assignment method implemented in NewHybrids^58^, despite the very different assumptions and algorithms the two approaches rely on^55,58^.

Though anthropogenic hybridization has been deeply investigated for a number of animal species, only a few studies applied reliable statistical criteria to define adequate assignment *q*-thresholds to correctly identify non-admixed individuals and distinguish different admixture classes^[1,41,42,73,77^. Conversely, most genetic investigations about hybridization in canids were mainly based on *q*-thresholds selected arbitrarily or chosen among those widely used in the literature (e.g. Malde *et al*.^41^) and rarely using simulated data to estimate error rates associated to the choice of a certain threshold^31,33,37,66,68,74^. A third challenge is thus represented by the adoption of objective criteria based on a Performance Analysis^38^ for setting the most appropriate *q*-thresholds to classify individuals into different admixture classes (e.g. pure *vs*. older admixed *vs*. recent admixed individuals) that could result into different management categories (e.g. operational pure, introgressed and operational hybrid individuals), minimizing the risk of both type I (pure individuals erroneously identified as admixed animals) and type II (admixed individuals falsely identified as pure animals) errors^5,12,16,33,38^.

Analysing the 39-STR marker panel, our assignment values appeared strongly robust even when introducing increasingly high levels of allelic dropout and missing data, nonetheless we remind that stringent filters on the quality and reliability of multilocus genotypes are essential to avoid significant biases in all downstream analyses. Our first selected *q-*threshold allowed us to correctly classify as admixed 100% of F1, F2, BC1W and 71% of BC2W, without any type I error. The remaining 29% of BC2W were classified as pure individuals likely due to a combination of: (i) higher mean *q*_iw_, closer to the identified *q*-threshold (0.955) compared to earlier generations of backcrossing (F1, F2, and BC1W), and (ii) wider CI compared to further generations of backcrossing (BC3W, BC4W, etc.).

Further backcrossing categories showed increasing percentages of assignment as pure individuals (40% in BC3W and 76% in BC4W), clearly showing the limits of the method in our study system when dealing with older backcrossing generations.

Nonetheless, the second empirical *q*-threshold allowed us to reliably discriminate also between real pure wolves and older admixed individuals, that only show a marginal dog ancestry and possibly deserve additional investigations.

Our results agree with other hybridization studies based on a comparable number of microsatellites, which highlighted the difficulty to reliably detect individuals with a domestic ancestry tracing back to more than two-three generations in the past^5,31,33,42,72,78^.

When the selected *q*-thresholds obtained with the 39-STR panel were applied to a large sample (*c*. 600 genotypes) of putative free-living wolves collected in Italy during the last 20 years, 73.8% of the analysed genotypes resulted operational pure animals (i.e. without relevant signs of domestic ancestor), while 13.5% were classifiable as introgressed individuals and 12.7% as operational hybrids, compatible with multiple and recurrent admixture events that might have occurred trough time, mostly during the phase of population re-expansion^26,31,33^. However, as shown by simulated data and confirmed by the genetic information derived from the analysis of the uniparental and coding markers, the operational pure category might include a proportion (in our case, 5.8%) of older admixed individuals not reliably detectable using the applied set of molecular markers.

Nonetheless, these percentages of admixed individuals cannot be intended as estimates of prevalence of admixed individuals in the Italian wolf population because the analysed samples had not been randomly collected, but mostly derived from specific monitoring projects focused on hybrid detection and from heterogeneously monitored areas^26,31,33,52,56^. Conversely, reliable estimates of hybridization prevalence could be assessed through statistical multi-event models applied to capture-recapture data obtained from well-planned long-term genetic and camera-trapping monitoring projects carried out through the entire Italian wolf distribution range^79–81^.

Despite 39 STRs represent a very limited portion of the genetic makeup of the analysed individuals that could be routinely applied to wide monitoring programs, the assignment values of recently-admixed individuals well correlate with those obtained from thousands of genome-wide markers^26^.

From a management perspective, known limits and efficiency in identifying different admixture classes allow to conceive corresponding management categories as robust as possible. However, a complication in the management of hybrids and backcrosses arises from the use of ambiguous or imprecise terminologies for defining different classes of admixed individuals. Therefore, in this study, we propose to categorize admixed individuals on the basis of empirically-defined *q*-thresholds, where “operational hybrids” correspond to recent admixed individuals (that include F1-F2 hybrids and most of the first two generations of backcrosses), while “operational pure individuals” correspond either to pure wolves or to older admixed individuals that could not be reliably distinguished from pure ones with the applied panel of molecular markers, but may retain marginal dog ancestry. Between them, we proposed an intermediate assignment class which mostly includes older admixed individuals that cannot be considered as operational pure animals, but do not require priority management actions given their limited domestic ancestry.

Given that hybridization should be primarily counteracted by (i) preventive measures aimed at reducing the number of free-ranging dogs, and (ii) proactive strategies to preserve prey availability, social cohesion, structure and connectivity of wolf packs, since habitat loss, rapid pack turnovers and recent population expansions are known to favor hybridization^82^, the proposed categorization would permit to avoid management interventions on pure animals erroneously classified as admixed individuals and their negative effects on the genetic and demographic viability of small or threatened wild populations^26,47,49,50^. Moreover, this categorization would allow to better focus efforts and resources toward “operational hybrids”, which carry significant portions of domestic genome ancestry and likely belong to the first generations of admixture, more efficiently than without any prioritization (e.g. genetically speaking, the removal of one hybrid with 50% dog ancestry would equal to the biological removal of 10 admixed individuals with 5% dog ancestry).

However, in those cases where an active management on operational hybrids is needed, the social acceptability of the applicable methods should be carefully considered, possibly avoiding controversial interventions such as lethal removal^3,16,82^. Indeed, among other more acceptable management methods, life-long captivation in welfare-respectful structures or sterilization and release of admixed individuals might represent feasible mitigation strategies^16,23^.

On the other side, the active management of introgressed individuals might become a necessary option where they locally occur at a high prevalence (that can be sometimes much higher than region- or population-wide estimates), thus increasing the probability of interbreeding between hybrids and retaining domestic variants on the long term^81,82^.

Conversely, dog-derived phenotypic traits, though validated by robust phenotype-genotype association tests^26^, when found in operational pure individuals should not be considered sufficient reasons for any intervention, since they might reflect old introgression events. Nonetheless they could represent useful clues for identifying potential hybrids with preliminary field surveying methods, such as camera trapping^79,80,83^, to be followed by further careful genetic investigations.

These classes appear to be more suitable for practical and management purposes compared to categories based on the supposed hybrid generations that, unless they are formally estimated based on genome-wide data^26^, are largely hazardous since a virtually infinite number of hybrid classes exists, with individual membership proportions widely overlapping.

These findings, together with the results derived from the analyses performed with our 12-STR marker panel, suggest that reduced molecular marker sets and empirical assignment *q*-thresholds can represent an effective first approach to orientate the most appropriate management actions.

Moreover, the recent possibility to access genome-wide SNP data to investigate anthropogenic hybridisation in a number of *taxa*^7,41,61^, including canids^24,26,44,77^, allows to gain a better resolution on the domestic ancestry proportions and to infer the real generations since the hybridization events^26,64,84^, that could be needed for the discrimination between real pure and older admixed individuals. Subsequently, the selection of reduced panels of ancestry-informative SNPs, including both neutral and coding mutations^26^, diagnosable by quantitative or microfluidic PCR techniques^77,85–87^, could be particularly suitable for cost-effective future monitoring projects based on the genotyping of invasive and non-invasive samples to be collected with a standardized design in hybridization hot-spots.

Our workflow, though designed on the case-study of the Italian wolf population, could be easily adapted to monitor the status of other populations and species potentially threatened by anthropogenic hybridization, although each study should adopt *ad-hoc q*-thresholds, based on the genetic distance between wild and domestic reference populations, their genetic diversity and possible substructure, but also on the number and type of analysed molecular markers. Moreover, when gene flow is known to occur between multiple wild populations (e.g. in Northeastern Alps and Carpathian Mountains^88–90^), the number of reference populations and the optimal number of genetic clusters *K* should be modified accordingly, in order to avoid the identification of false wild *x* domestic hybrids (type I errors). Nonetheless, we also remind that such complex systems also require large parental populations to be used as reference. Of course, such an effort is worth using only when dealing with complex levels of admixture, whereas for simpler systems (e.g. when a few individuals could be assigned to recent crosses (F1, F2) or backcrosses (BC1)) standard approaches are sufficient.

In conclusion, the identification of operational categories based on admixture classes outlined through simulations can support scientists, practitioners and decision-makers in the implementation of more efficient conservation strategies mostly focusing on recent hybrids, whose diffusion and consequent spread of domestic alleles could be limited by active management actions to be defined upon local context and acceptance levels toward the presence of free-ranging admixed individuals, but taking into account that nonlethal actions such as captivation or sterilization are often considered by scientists and the public opinion as more feasible and ethically acceptable conservation tools^16^.

## Materials and Methods

### Ethical statements

No ethics permit was required for this study, and no animal research ethics committee prospectively was needed to approve this research or grant a formal waiver of ethics approval since the collection of wolf samples involved dead animals. Fieldwork procedures were specifically approved by ISPRA as a part of national wolf monitoring activities.

Dog blood samples were collected by veterinarians during health examinations with a not-written (verbal) consent of their owners (students/National park volunteers/or specialised technician personnel of the Italian Forestry Authority (CFS)), since they were interested on wolf conservation studies and monitoring projects in Italy. Moreover there is not a relevant local law/legislation that exempts our study from this requirement.

Additionally no anesthesia, euthanasia, or any kind of animal sacrifice was applied for this study and all blood samples were obtained aiming at minimizing the animal suffering.

### Selection of the reference populations

Reference wild parentals were selected from found-dead wolves collected across the Italian peninsular distribution range that showed the typical wild coat colour pattern and no other apparent dog-like traits such as white claws or spurs on the hind legs^26,31,33,91^.

Reference domestic parentals were selected from free-ranging mongrels and village dogs sampled in the same areas of the reference wolves, plus one male and one female randomly chosen from 14 wolf-sized dog breeds. Given the high between-breed variation^92^ these samples could represent a good proxy of the diversity in dogs while avoiding significant sub-structuring during clustering analyses^26,33,47,93^.

As wild and domestic reference individuals, all available in the ISPRA canid database^26,33,52,56^, we only retained those whose genotypes showed no missing data and proportions of membership *q*_i_ > 0.990 to the respective wild or domestic clusters estimated in previous Bayesian assignment procedures performed, using the software Structure v.2.3.4^57,94^, on 39 canine STRs commonly used to reconstruct individual genotypes in some of the most recent studies on wolf *x* dog hybridization in Europe^26,33,47,53^. This conservative *q*-threshold was selected to avoid the inclusion of older admixed individuals among the wild reference population, thus reducing the power to correctly identify admixed individuals in the tested dataset. Furthermore, 90 of the selected reference wolves and 30 of the selected reference dogs were also tested in Maximum-Likelihood assignment procedures performed analysing 156 K genome-wide canine SNPs in the software Admixture v.1.23^95^ and confirmed their pure status showing *q*_i_ > 0.990^26^.

### Simulation of pure and admixed populations

Reference samples were used in HybridLab^54^ to simulate 100 genotypes (a sufficient number to well represent the parental allele frequencies^40^) for each of the following pure and admixed classes: wild (PW) and domestic (PD) parentals, first (F1) and second (F2) generation hybrids, and eight backcross generations (BC1W-BC8W) with wild parentals (Supplementary Table S1b). In a selectively neutral perspective, BC8W individuals should theoretically retain less than 0.2% of the domestic parental ancestry (Supplementary Table S1b). Simulations were performed both with the complete set of 39 STRs^33,47^ and with a reduced set of 12 STRs commonly utilized for genotyping low-content DNA samples through a multiple-tube approach in non-invasive genetic monitoring projects^52,56^.

### Bayesian assignment tests

To perform admixture analyses and assign individuals to their reference populations, empirical and simulated multilocus microsatellite genotypes were run using the R package Parallel Structure^55^, which uses the back-end executable of Structure^57,94^ parallelizing the Markov Chain Monte Carlo (MCMC) algorithm to: (i) distribute computation jobs among multiple processors, thus speeding up analysis times, and: (ii) automatically subdivide a dataset of genotypes to be assigned to predefined reference populations into multiple single projects (each project is composed by the reference populations and one of the genotypes to be assigned) which are independently run, preventing that sample sizes or the simultaneous analysis of samples with different levels of admixture might affect results^69,70^. Custom bash and excel macro scripts were designed to assembly output files, that are equal to the number of the analysed samples, and to create a single summary result file (See the Supplementary Fig. S1, the Supplementary Text S2 and the Supplementary Table S7 for the detailed pipeline).

We ran four independent replicates of Parallel Structure with 5 × 10^5^ iterations following a burn-in period of 5 × 10^4^ iterations, using the *Admixture* (*A*) and *Independent allele frequencies* (*I*) models, which are the most suitable ones to investigate gene flow between populations with reasonably different allele frequencies and independently evolving^66,94^, and assuming *K* = 2 *a priori* clusters (corresponding to the optimal number of genetic clusters in which reference populations are split to identify the proportion of admixture^33,52^). For each group, we assessed the average proportion of membership (*Q*_i_) to the two clusters and individual assignments were based on proportions of membership (*q*_i_) estimated for every single individual. We also estimated 90% credibility intervals (CI) for both *Q*_i_ and *q*_i_ in order to evaluate their overlap between different admixture categories and their individual width, expecting wider CI in the assignment of admixed individuals due to difficulties in estimating parental allele frequencies^57,66,94^.

In order to test the robustness of the assignment values under varying levels of genotyping errors and missing data, we simulated increasing levels of allelic dropout (ADO) and missing data (number of missing loci) for the 1200 simulated parental and admixed genotypes (both at 39 and 12 STRs) in Gimlet 1.3.3^96^, assuming 10%, 20% and 30% for both parameters, then re-ran the assignment tests in Parallel Structure^55^ with the same settings.

The software NewHybrids^58^ was used to compute the posterior probabilities that each genotype belongs to each of the following five classes: wild and domestic parentals (PW and PD), first (F1) and second (F2) generation hybrids, and first backcrosses of F1 with wolves (BC1W). Posterior distributions were evaluated running four independent replicates of NewHybrids^58^ with 10^5^ iterations of the Monte Carlo Markov chains, following a burn-in period of 10^4^ iterations, without any individual or allele frequency prior information, and using “*Jeffreys-like*” or “*Uniform*” priors for both mixing proportions and allele frequencies^58^.

### Criteria for the definition of admixture thresholds and assignment error rates

We tried to identify the most appropriate *q*-thresholds that were able to distinguish between pure, older admixed and recent admixed individuals (Table 1), while minimizing the risk of both type I (actually pure individuals erroneously identified as admixed animals) and type II (admixed individuals falsely identified as pure animals) errors^12,16,26,33^.

Therefore, we estimated the “performance” of different *q*-thresholds with intervals of 0.005, spanning from 0.500 to 0.999. In particular, each performance was computed as the product between the “mean efficiency”, which is the ratio of the number of admixed individuals correctly identified on the total number of admixed individuals actually included in the sample, and the “accuracy”, defined as the number of admixed individuals correctly assigned to a certain simulated admixture class on the total number of individuals actually belonging to that class^38^.

Each *q*-threshold was tested between groups of simulated individuals at increasing levels of admixture (e.g. PW *vs*. BC8W to BCW1, F2 & F1; PW & BC8W *vs*. BC7W to BCW1, F2 & F1; PW, BC8W & BC7W *vs*. BC6W to BCW1, F2 & F1; PW, BC8W, BC7W & BC6W *vs*. BC5W to BCW1, F2 & F1; PW, BC8W, BC7W, BC6W & BC5W *vs*. BC4W to BCW1, F2 & F1, etc.), considering a minimum performance of 0.90 for any combination to be retained^38^.

### Application of the identified admixture thresholds to the management classification of tested samples

The selected *q*-thresholds were finally applied to classify the 39-STR canid genotypes obtained from the carcasses of 569 putative wolves (236 females and 333 males) collected from 1987 to 2019 throughout the whole wolf distribution range in Italy^26,33,47,52^. Extraction, amplification and post-amplification procedures were carried out in separate rooms reserved to low-template DNA samples following protocols described in Randi *et al*.^33^, Fabbri *et al*.^52^ and Caniglia *et al*.^56^. To check for the occurrence of allelic dropout and false alleles, samples were independently analysed twice for each locus. Negative (no DNA) and positive (samples with known genotypes) PCR controls were used to check for laboratory contaminations. Genotypes were accepted as reliable only when ADO and missing data were less than 10%^33,52,56^.

Assignments of the 39-STR canid genotypes were further integrated with the information derived from uniparental markers (mtDNA control region and four Y-linked STRs) and from the functional melanistic deletion at the β-defensin CBD103 gene (corresponding to the *K-*locus), which were used to provide the directionality of the hybridization and determine the presence of the atypical dog-derived black coat coloration^18,31,33,52^.

Based on the assignment results, both simulated (pure and admixed) and real 39-STR canid genotypes were classified into three appropriate management classes (Table 1): “operational pure individuals” (including pure wolves and admixed individuals with a negligible dog ancestry, that do not require management actions), “introgressed individuals” (likely old admixed individuals with a marginal domestic ancestry that only require low priority management actions, such as further investigations) and “operational hybrids” (recent admixed individuals with a clearly detectable dog ancestry, that should be targeted by high priority management operations such as sterilization or captivation).

## Supplementary information

Supplementary Information.

Supplementary Table S1-S6

Supplementary Table S7

## Data Availability

The majority of the data generated and analysed during the current study are presented within the published article or in Supplementary information files. The raw data are available from the corresponding author on reasonable request.
