# Supplementary material for: A standardized approach to empirically define reliable assignment thresholds and appropriate management categories in deeply introgressed populations
Source: Sci Rep. 2020 Feb 18;10:2862. doi: 10.1038/s41598-020-59521-2 (PMC7028925; doi:10.1038/s41598-020-59521-2)
Supplement: Supplementary file 1 — Supplementary Information. [file 41598_2020_59521_MOESM1_ESM.docx]

**A standardized approach to empirically define reliable assignment thresholds and appropriate management categories in deeply introgressed populations**

**Romolo Caniglia^1#^, Marco Galaverni^2^, Edoardo Velli^1^, Federica Mattucci^1^, Antonio Canu^3^, Marco Apollonio^3^, Nadia Mucci^1^, Massimo Scandura^3^, Elena Fabbri^1^**

^1^Unit for Conservation Genetics (BIO-CGE), Italian Institute for Environmental Protection and Research (ISPRA), Ozzano dell’ Emilia, Bologna, Italy

^2^Conservation Unit, WWF Italia, Rome, Italy

^3^Department of Veterinary Medicine, University of Sassari, Sassari, Italy

**^#^**Correspondence and requests for materials should be addressed to: [romolo.caniglia@isprambiente.it](mailto:romolo.caniglia@isprambiente.it)

**Supplementary Information**

**Supplementary Text S1**

**Bayesian assignment tests and *q*-threshold selection procedures performed with the 12-STR marker panel**

**Membership proportions and individual coefficients from the assignment tests**

Results of the assignment analyses obtained using the reduced marker panel of 12 STRs in Parallel Structure^1^ showed that all reference parental genotypes were fully assigned to their clusters (Supplementary Table S4) with an average membership proportion *Q*_wolf_ = 0.997 (CI = 0.989-1.000) and *Q*_dog_ = 0.997 (CI = 0.989-1.000), and *q*_iw_ ranging from 0.990 to 0.999 and *q*_id_ ranging from 0.990 to 0.999 (Supplementary Table S4).

The wild and domestic simulated parental populations showed *Q*_i_ and *q*_i_ values almost completely overlapping with those of the wild and domestic reference populations, and were assigned to their respective clusters with *q*_iw_ ≥ 0.995 for the wild and *q*_id_ ≥ 0.990 for the domestic parentals (Supplementary Fig. S3a).

First generation (F1) hybrids showed intermediate *Q*_i_ and *q*_i_ values (Supplementary Table S4 and Supplementary Fig. S3a). Conversely, F2 hybrids showed the widest range of *q*_iw_ values ranging from 0.143 to 0.971. As expected, and confirmed also by the width of CI in the different admixed classes, the 12-STR panel provided a lower resolution in detecting backcrosses than the 39-STR panel, indeed, 4% of BC1W already showed *q*_iw_ overlapping those of the wild parental population (Supplementary Table S4 and Supplementary Fig. S3a).

**Selection and performance of the appropriate *q*-thresholds**

Accuracy, efficiency and performance of the reduced 12-STR marker panel were calculated for different candidate *q*-thresholds ranging from 0.500 to 0.999 (Supplementary Table S5 and Supplementary Fig. S4).

A performance higher than 0.80 (a value that we considered to be sufficiently reliable for a reduced number of molecular markers such as the one considered in this study and commonly used in wolf non-invasive genetic monitoring projects^2-4^) was obtained for three category combinations (Supplementary Fig. S4): PW & BCW8 to BCW1 *vs.* F1 & F2 (performance = 0.903 at *q*-threshold = 0.710), PW & BCW8 to BCW2 *vs.* BC1W, F2 & F1 (performance = 0.858 at *q*-threshold = 0.875) and PW & BCW8 to BCW3 *vs.* BC2W, BC1W, F2 & F1 (performance = 0.822 at *q*-thresholds = 0.975).

However, the percentages of the simulated admixed individuals which were identified as assignment-pure animals considerably decreased at increasing values of the *q*-thresholds: 1% F1, 7% F2, 77% BC1W, 97% BC2W at the *q*-threshold = 0.710; 0% F1, 1% F2, 35% BC1W and 76% BC2W at the *q*-threshold = 0.875; 0% F1, 0% F2, 14% BC1W and 52% BC2W at the *q*-threshold = 0.975 (Supplementary Table S6).

Therefore, to be as much conservative as possible, we decided to retain the *q*-threshold value of 0.975 to more efficiently discriminate between recent admixed individuals (F1-BC2W) and all the other simulated classes including older admixed (from BC3W onwards) plus pure (PW) individuals. At the selected threshold, indeed, 100% of the wolf *x* dog F1 and F2, 86% of BC1W and 48% of BC2W were coherently classified as recent admixed individuals (Supplementary Table S6).

However, since none of the highly-performing *q*-thresholds was able to reliably discriminate between older admixed and pure individuals, we introduced a second *q*-threshold at 0.990, representing the minimum individual *q*_iw_ assignment value of the simulated and real wild parentals. Therefore, we assumed that the assignment interval in the range 0.975 and 0.990 can include older admixed individuals, showing only a marginal dog ancestry (< 5%). In this way, 8% of BC1W, 12% of BC2W were classified as older admixed individuals, together with 23% of BC3W, 15% of BC4W, 6% of BC5W, 2% of BC6W, 4% of BC7W and 2% of BC8W (Supplementary Table S6). Above this second *q*-threshold type I errors confirmed to be absent but type II errors were further minimized since we found 6% of BC1W 40% of BC2W, 64% BC3W, 77% BC4W, and more than 90% of BC5W-BC8W clustering together with reference and simulated wolf parentals (Supplementary Table S6).

The Bayesian assignment performed in NewHybrids^5^ also proved to be highly efficient showing that all wild and domestic reference and all wild and domestic simulated parentals had the best posterior probabilities (*P* ≥ 0.900) to be purebred animals. Interestingly, and as expected with a reduced number of markers, 62% F1, 45% F2 and 40% BC1W clearly assigned to their own categories (*P* ≥ 0.900) and none F1 nor F2 shared posterior probabilities to belong to wolves or dogs < 0.09 (Supplementary Fig. S3b). However, 10% BC1W, 43% BC2W, 84% BC3W and more than 90% BC4W showed significant posterior probabilities (*P* ≥ 0.900) to be classified as wolves.

When we applied the *q*-thresholds of 0.975 and 0.990, selected for the reduced marker panel, to the 12-STR genotypes of the real 569 putative wolves, 89.7% of the identified operational pure and 54.2% of operational hybrid individuals confirmed their management category also when their genotypes were extended at 39 STRs. Conversely, 13.9% of the operational hybrids identified using 12-STRs resulted operational pure animals, and 31.9% resulted introgressed individuals when analysed at 39 STRs. Therefore, these findings suggest that the reduced marker panel of 12 STRs, commonly used for wolf non-invasive genetic monitoring projects in Italy^2-4^, should be applied, together with other surveying methods such as camera trapping, only to preliminarily investigate areas of supposed wild *x* domestic hybridization^2,6-8^. If operative hybrids are identified, the capture of the wild-living animals in the explored territories should be recommended to analyse their DNA at the 39-STR marker panel and, based on the emerging results, proceed with the most appropriate management solutions.

**Supplementary Text S2**

**Custom bash and excel macro scripts designed to assembly Parallel Structure output files**

The software Parallel Structure^1^ allows to automatically subdivide a dataset of genotypes to assign to predefined reference populations into multiple single projects which are independently run, preventing that sample sizes or cohorts of genetically heterogeneous samples might affect results. Custom bash and excel macro scripts were designed to assembly output files, that are equal to the number of the analysed samples, and to create a single summary result file.

The Supplementary Table S7 allows to quickly assemble and read results obtained running the software Parallel Structure with *K* = 2.

To correctly install and use the R package “Parallel Structure” please refer to Besnier and Glover^1^. Based on our experience the software “Parallel Structure” does not properly work on Windows Operating System (OS) so we suggest to run it from a Unix-like OS (e.g. Ubuntu).

**Operative instructions to run Parallel Structure**

Create a file named “ind_list.txt” in which sample names are listed in a single column. Please, be sure that the End of Line characters are in Linux format.

Put the “ind_list.txt” file inside the folder containing results from Parallel Structure. If you asked for the "printqhat" result files please put the “results_job_*_f” files and the “ind_list.txt” file in a separate folder.

Open the “bash” prompt and set as working directory the folder in which you put the “results_job_*_f” and “ind_list.txt” files. If you are in a Windows OS you could use Cygwin or other GNU tools.

Run this commands: grep -w -h -B 1 -f ind_list.txt results_job_T* | sed 's/ \+/\t/g' | sed 's/\t$//' >> summary.txt.

In each output file produced by the software Parallel Structure, columns containing assignment *q*-values to a certain cluster (e.g. dog or wolf) are assigned pretty randomly.

To correctly sort and read the results you could use the excel sheet of Supplementary Table S7, copy the content of the “summary.txt” file and paste it in the corresponding cells of the Supplementary Table S7. Assignment *q*-values to a cluster are now consistently aligned all in the same column.

**References**

1. Besnier, F. & Glover, K. A. Parallel Structure: a R package to distribute parallel runs of the population genetics program Structure on multi-core computers. *PLoS One* **8**, e70651 (2013).
2. Galaverni, M., Palumbo, D., Fabbri, E., Caniglia, R., Greco, C. & Randi, E. Monitoring wolves (*Canis lupus*) by non-invasive genetics and camera trapping: a small-scale pilot study. *Eur. J. Wildl. Res.* **58**, 47-58 (2012).

3. Caniglia, R., Fabbri, E., Galaverni, M., Milanesi, P. & Randi, E. Noninvasive sampling and genetic variability, pack structure, and dynamics in an expanding wolf population. *J. Mammal.* **95**, 41-59 (2014).

4. Fabbri, E., Velli, E., D’Amico, F., Galaverni, M., Mastrogiuseppe, L., Mattucci, F. & Caniglia, R. From predation to management: monitoring wolf distribution and understanding depredation patterns from attacks on livestock. *Hystrix, Ital. J. Mammal.* **29**, 101-110 (2018).

5. Anderson, E. C. & Thompson, E. A. A model-based method for identifying species hybrids using multilocus genetic data. *Genetics* **160**, 1217-1229 (2002).

6. Canu, A., Mattioli, L., Santini, A., Apollonio, M. & Scandura, M. “Video-scats”: combining camera trapping and non-invasive genotyping to assess individual identity and hybrid status in gray wolf. *Wildllife Biol.* **4**, 1-10 (2017).

7. Mattioli, L., Canu, A., Passilongo, D., Scandura, M. & Apollonio, M. Estimation of pack density in grey wolf (*Canis lupus*) by applying spatially explicit capture-recapture models to camera trap data supported by genetic monitoring. *Front. Zool.* **15**, 38 (2018).

8. Salvatori, V., Godinho, R., Braschi, C., Boitani, L. & Ciucci, P. High levels of recent wolf *x* dog introgressive hybridization in agricultural landscapes of central Italy. *Eur. J. Wildl. Res.* **65**, 73, DOI:[10.1007/s10344-019-1313-3](https://doi.org/10.1007/s10344-019-1313-3) (2019).

**Supplementary Tables**

**Supplementary Table S1.** Glossary of the terms and corresponding definitions used in the paper, referred to **(a)** anthropogenic hybridization categories; and **(b)** to the simulated genotypes of wild (W) and domestic (D) parentals, first (F1) and second (F2) generation hybrids, and eight backcross generations (BC1W-BC8W) with wild parentals. For each simulated class of genotypes the corresponding domestic parental ancestry theoretically retained in a selectively neutral perspective is reported. Simulated wild (PW) and domestic (PD) parentals, first (F1) and second (F2) generation hybrids, and eight backcross generations (BC1W-BC8W) with wild parentals.

**Supplementary Table S2.** Accuracy, efficiency and performance calculated for different candidate *q*-thresholds ranging from 0.500 to 0.999 for the 39-STR marker panel. Data comprise the 39-STR genotypes of the simulated wild (PW) and domestic (PD) parentals, first (F1) and second (F2) generation hybrids, and eight backcross generations (BC1W-BC8W) with wild parentals.

**Supplementary Table S3.** Average posterior probabilities to belong to the genotype classes of domestic and wild parentals (PD and PW), first (F1) and second (F2) generation hybrids, and eight backcross generations (BC1W-BC8W) with wolves (form BC1W to BC8W) as inferred through the Bayesian assignment analyses of the 39-STR reference and simulated genotypes performed in NewHybrids using the “*Jeffreys-like*” priors.

Data comprise the 39-STR genotypes of the reference wild (RW) and reference domestic (RD) individuals, simulated wild (PW) and domestic (PD) parentals, first (F1) and second (F2) generation hybrids, and eight backcross generations (BC1W-BC8W) with wild parentals.

**Supplementary Table S4.** Average membership proportions *Q*_i_ to the wolf (*Q*_wolf_) or dog (*Q*_dog_) cluster and their credibility intervals (90% CI) estimated through the Bayesian assignment analyses of the 12-STR reference and simulated genotypes performed in Parallel Structure, assuming

*K* = 2 clusters and using the ‘‘*Admixture*’’ and ‘‘*Independent allele frequencies*’’ models. Data comprise the 12-STR genotypes of the reference wild (RW) and reference domestic (RD) individuals, simulated wild (PW) and domestic (PD) parentals, first (F1) and second (F2) generation hybrids, and eight backcross generations (BC1W-BC8W) with wild parentals.

**Supplementary Table S5.** Accuracy, efficiency and performance calculated for different candidate *q*-thresholds ranging from 0.500 to 0.999 and for the 12-STR marker panel. Data comprise the 12-STR genotypes of the simulated wild (PW) and domestic (PD) parentals, first (F1) and second (F2) generation hybrids, and eight backcross generations (BC1W-BC8W) with wild parentals.

**Supplementary Table S6.** Proportions of real and simulated 12-STR genotypes correctly identified as assignment pure, older admixed and recent admixed individuals and, consequently classifiable as operational pure, introgressed and operational hybrid individuals using the two selected *q*-thresholds (0.990, representing the minimum individual *q*_iw_ assignment value of the simulated and real wild parentals, and 0.975, selected on the basis of the performance analysis) which, minimizing the risk of both type I and type II errors, are able to efficiently discriminate between the three proposed assignment classes and corresponding management categories. Bayesian analyses were performed in Parallel Structure, assuming *K* = 2 clusters and using the ‘‘*Admixture*’’ and ‘‘*Independent allele frequencies*’’ models. Data comprise the 12-STR genotypes of the reference wild individuals (RW), simulated wild parentals (PW), first (F1) and second (F2) generation hybrids, and eight backcross generations (BC1W-BC8W) with wild parentals.

**Supplementary Table S7.** Excel macro scripts designed to assembly Parallel Structure output files.

**Supplementary Figures**

**
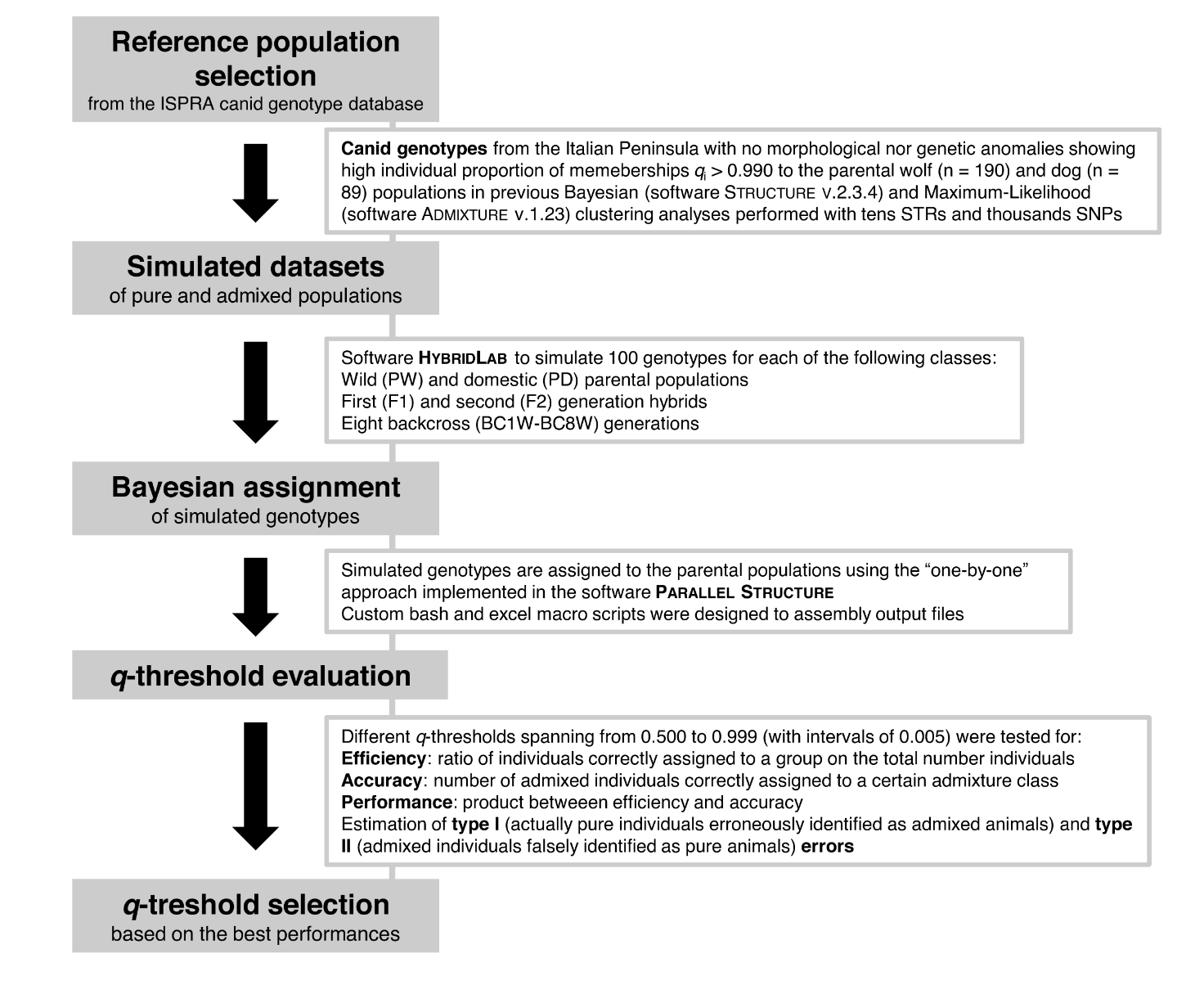
**

**Supplementary Figure S1.** Workflow describing the selection of the reference wolf and dog parental populations and the simulations of parental, hybrid and backcross genotype classes to run Bayesian assignment analyses and define adequate and reliable *q-*thresholds to classify individuals into discrete levels of domestic ancestry. The gray boxes describe the main workflow steps whereas the white boxes summary the different criteria used to develop the relative upper steps.

**
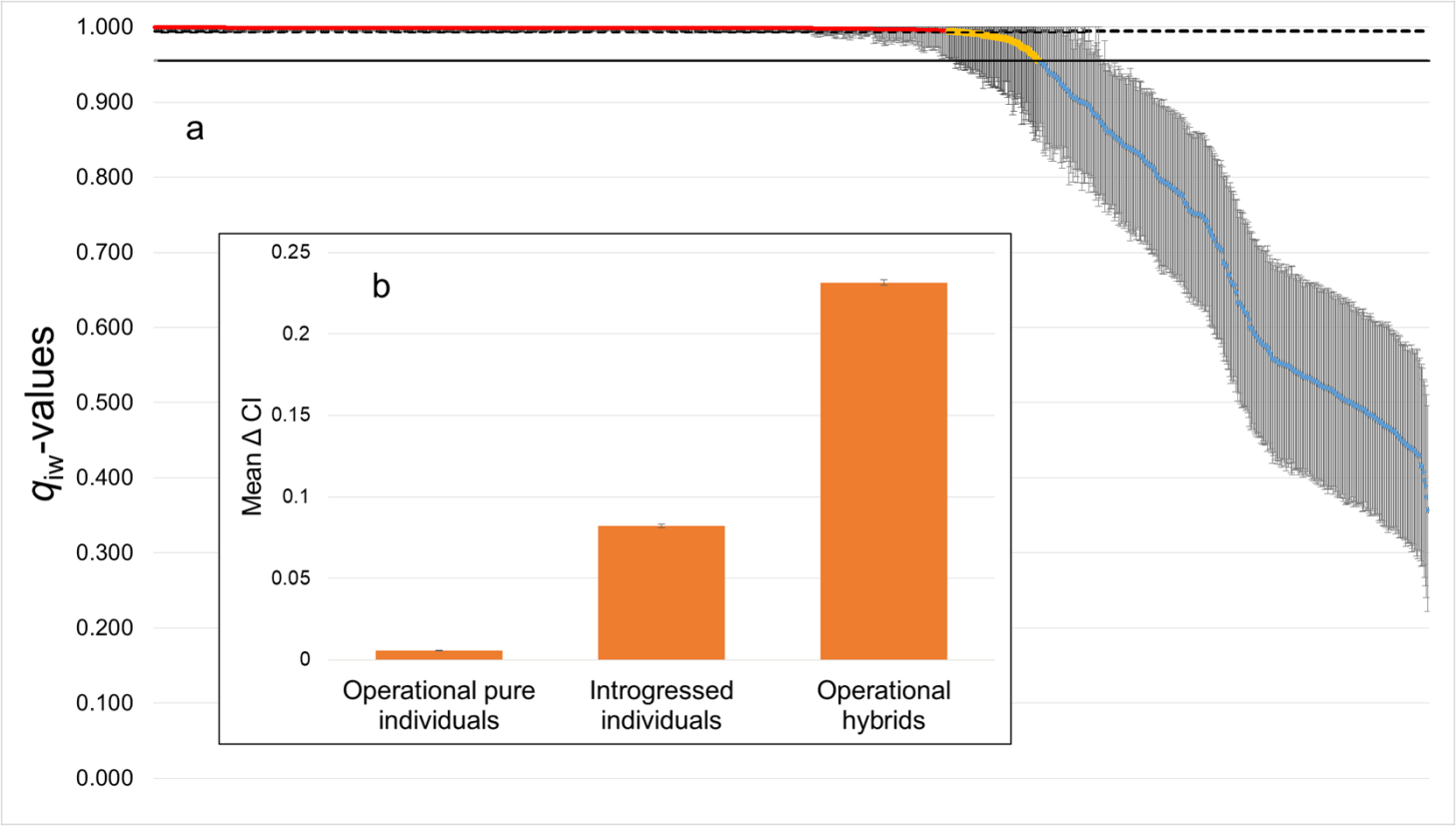
**

**Supplementary Figure S2. (a)** Distribution of the individual *q*_iw_-values (colored dots) and their 90% credibility intervals (CI; vertical grey whiskers) of the reference (RW) and parental (PW) wolves, first (F1) and second (F2) generation hybrids, and eight backcross (BC1W-BC8W) generations to the wild parentals obtained running the real and simulated 39-STR genotypes in the software Parallel Structure, assuming *K* = 2 clusters and using the ‘‘*Admixture*’’ and ‘‘*Independent allele frequencies*’’ models. Red, yellow and blue dots indicate, respectively, *q*_iw_-values and corresponding CI associated to the genotypes assigned as pure, older admixed and recent admixed individuals and, consequently, classifiable as operational pure, introgressed and operational hybrid individuals, applying the two selected *q*-thresholds (0.995, indicated by the dashed line and representing the minimum individual *q*_iw_ assignment value of the simulated and real wild parentals, and 0.955, indicated by the solid line and selected on the basis of the performance analysis). (**b)** Histogram of the average widths (ΔCI) of the 90% confidential intervals (CI) and corresponding standard errors (vertical grey whiskers) associated to the 39-STR genotypes assigned, on the basis of the two selected *q*-thresholds, as pure, older admixed and recent admixed individuals and, consequently, classifiable as operational pure, introgressed and operational hybrid individuals.

**
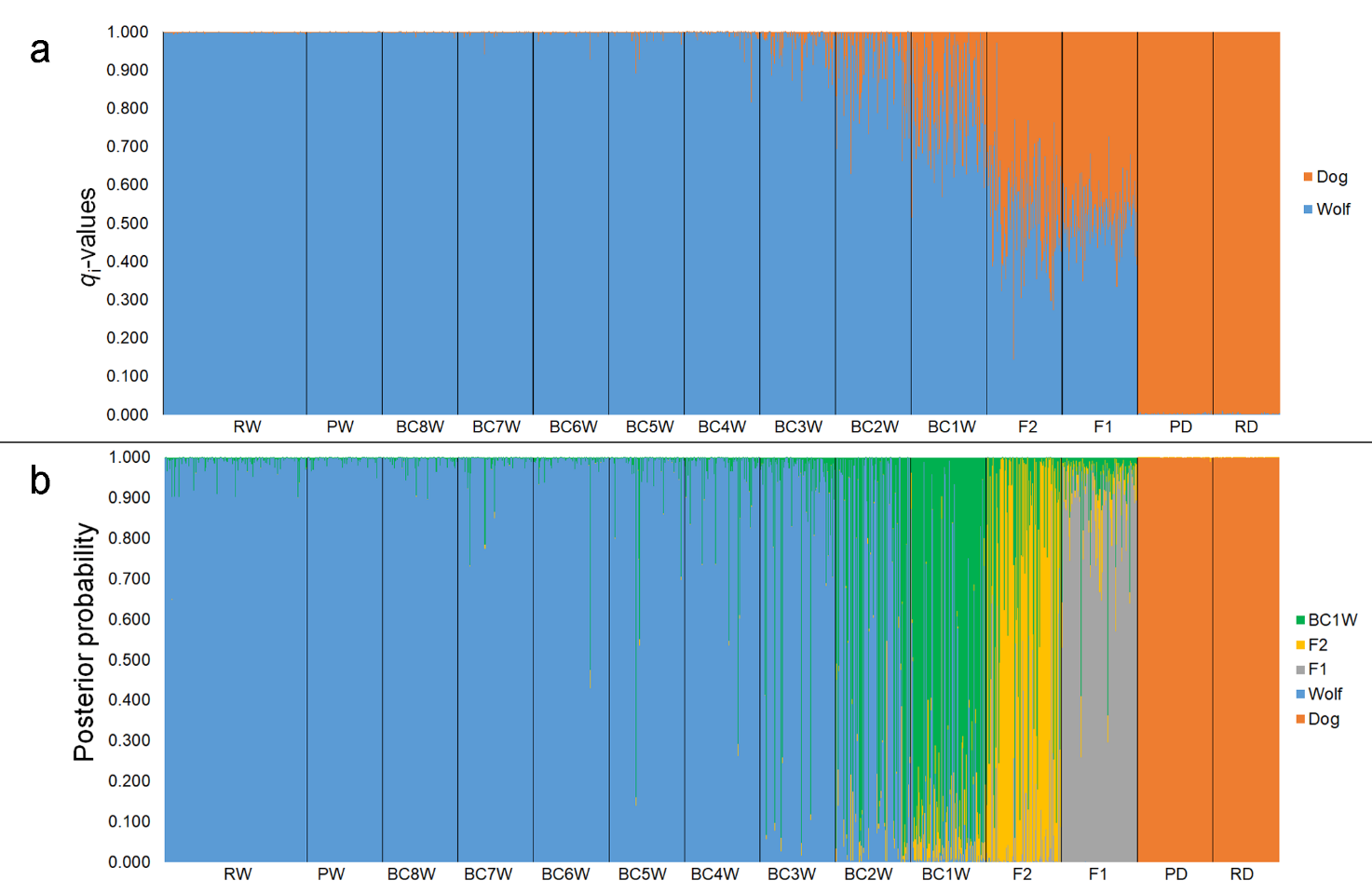
**

**Supplementary Figure S3. (a)** Bar plotting of the individual *q*_i_-values obtained assigning the 12-STR genotypes of the simulated wild (PW) and domestic (PD) parentals, first (F1) and second (F2) generation hybrids, and eight backcross generations (BC1W-BC8W) with wild parentals to the wolf (RW) and dog (RD) reference populations. Each individual is represented by a vertical line partitioned into colored segments, whose length is proportional to the individual coefficients of membership (*q*_i_) to the wolf and dog clusters inferred by a Bayesian assignment analyses performed by the software Parallel Structure, assuming *K* = 2 clusters and using the ‘‘*Admixture*’’ and ‘‘*Independent allele frequencies*’’ models. **(b)** Posterior probabilities estimated, for the 12-STR genotypes of the simulated wild (PW) and domestic (PD) parentals, first (F1) and second (F2) generation hybrids, and eight backcross generations (BC1W-BC8W) with wild parentals to the wolf (RW) and dog (RD) reference populations, using the software NewHybrids with the “*Jeffreys-like*” priors for both mixing proportions and allele frequencies. Each individual is represented by a horizontal bar divided in five segments corresponding to its probability to belong to five genotypic classes: wild and domestic parentals (PW and PD), F1, F2, and first backcrosses of F1 with wolves (BC1W).

**
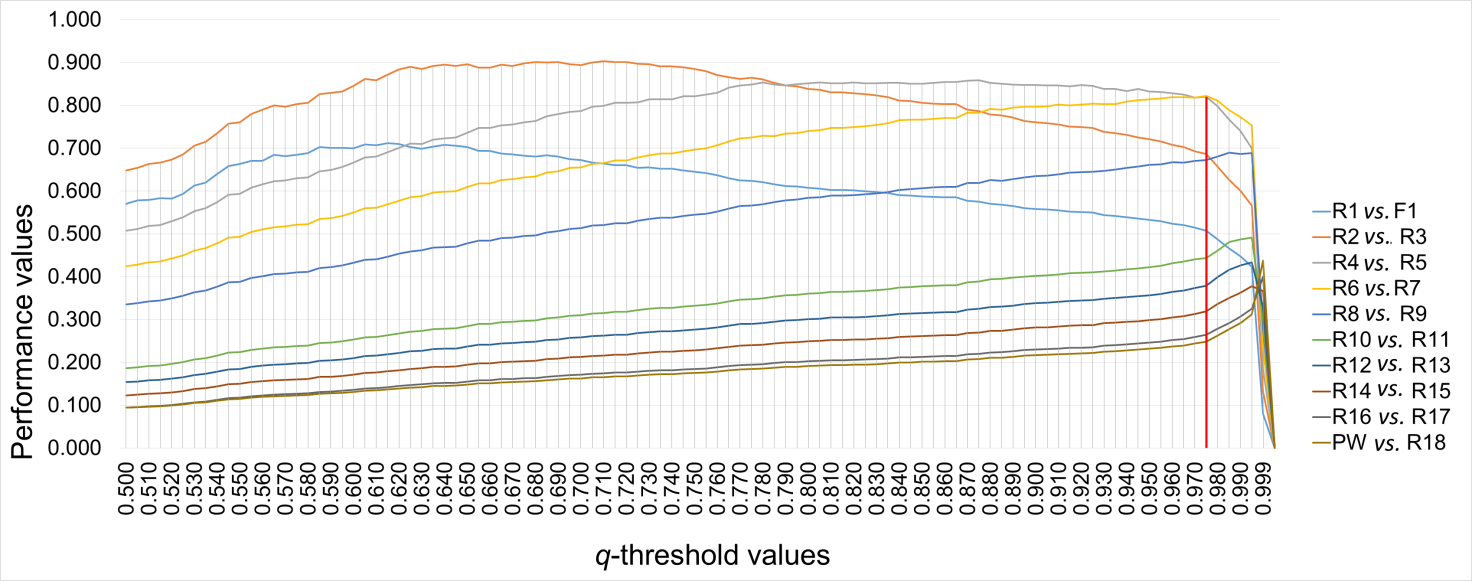
**

**Supplementary Figure S4.** Graphical trends of the average performances (on the y-axis) estimated for increasing values of *q*-thresholds (on the x-axis). Each performance was computed as the product between the mean efficiency (the ratio of the number of admixed individuals correctly identified on the total number of admixed individuals actually included in the sample) and the accuracy (the number of admixed individuals correctly assigned to a certain admixture class on the total number of individuals actually belonging to that class) obtained considering individual *q*_i_ values of the simulated 12-STR genotypes estimated from the Bayesian assignment analyses performed in Parallel Structure, assuming *K* = 2 clusters and using the ‘‘*Admixture*’’ and ‘‘*Independent allele frequencies*’’ models. Each *q*-threshold was tested considering comparisons between groups (**Rn**) including simulated individuals for increasing levels of admixture. **R1**: PW, BC8W to BC1W & F2; **R2**: PW, BC8W to BC1W; **R3**: F1 & F2; **R4**: PW, BC8W to BC2W; **R5**: BC1W, F2 & F1; **R6**: PW, BC8W to BC3W; **R7**: BC2W to BC1W, F2 & F1; **R8**: PW, BC8W to BC4W; **R9**: BC3W to BC1W, F2 & F1; **R10**: PW, BC8W to BC5W; **R11**: BC4W to BC1W, F2 & F1; **R12**: PW, BC8W to BC6W; **R13**: BC5W to BC1W, F2 & F1; **R14**: PW, BC8W to BC7W; **R15**: BC6W to BC1W, F2 & F1; **R16**: PW & BC8W; **R17**: BC7W to BC1W, F2 & F1; **R18**: BC8W to BC1W, F2 & F1. The vertical red line identifies the *q*-threshold (*q*_i_ *=* 0.975) selected on the basis of the performance analysis carried out comparing **R6** *vs.* **R7** (yellow line).
